# Ligand-Induced Modulation of the Free-Energy Landscape of G Protein-Coupled Receptors Explored by Adaptive Biasing Techniques

**DOI:** 10.1371/journal.pcbi.1002193

**Published:** 2011-10-13

**Authors:** Davide Provasi, Marta Camacho Artacho, Ana Negri, Juan Carlos Mobarec, Marta Filizola

**Affiliations:** Department of Structural and Chemical Biology, Mount Sinai School of Medicine, New York, New York, United States of America; National Cancer Institute, United States of America and Tel Aviv University, Israel

## Abstract

Extensive experimental information supports the formation of ligand-specific conformations of G protein-coupled receptors (GPCRs) as a possible molecular basis for their functional selectivity for signaling pathways. Taking advantage of the recently published inactive and active crystal structures of GPCRs, we have implemented an all-atom computational strategy that combines different adaptive biasing techniques to identify ligand-specific conformations along pre-determined activation pathways. Using the prototypic GPCR β2-adrenergic receptor as a suitable test case for validation, we show that ligands with different efficacies (either inverse agonists, neutral antagonists, or agonists) modulate the free-energy landscape of the receptor by shifting the conformational equilibrium towards active or inactive conformations depending on their elicited physiological response. Notably, we provide for the first time a quantitative description of the thermodynamics of the receptor in an explicit atomistic environment, which accounts for the receptor basal activity and the stabilization of different active-like states by differently potent agonists. Structural inspection of these metastable states reveals unique conformations of the receptor that may have been difficult to retrieve experimentally.

## Author Summary

G protein-coupled receptors (GPCRs) constitute one of the most important classes of cellular targets owing to their known response to a host of extracellular stimuli, and consequent involvement in numerous vital biological processes. Compelling evidence herein referred to as ‘functional selectivity’ shows that ligands with varied efficacies can stabilize different GPCR conformations that may selectively interact with different intracellular proteins, and therefore induce different biological responses. Understanding how this selectivity is achieved may lead to the discovery of drugs with improved therapeutic properties. We propose here a computational strategy that enables identification of the specific conformations assumed by a GPCR when interacting with ligands that elicit different physiological responses. Not only can these computational models help bridge the information gap in structural biology of GPCRs, but they can be used for virtual screening, and possibly lead to the structure-based rational discovery of novel ‘biased’ ligands that are capable of selectively activating one cellular signaling pathway over another.

## Introduction

G-protein coupled receptors (GPCRs) are versatile signaling proteins that functionally couple a host of extracellular stimuli to intracellular effectors, thus mediating several vital cellular responses. The majority of marketed drugs act as agonists, inverse agonists, or antagonists at these receptors depending on whether they increase, reduce, or have no effect on the so-called ‘basal activity’ that characterizes unliganded GPCRs for diffusible ligands. Not only can a specific GPCR activate different G-protein or arrestin isoforms [1], but a single ligand can display different efficacy for different signaling pathways, an observation that has been dubbed “functional selectivity”, “agonist trafficking”, “biased agonism”, “differential engagement”, or “protean agonism” in the literature [2]–[6].

At the molecular level, a simple explanation for this phenomenon is that ligands with varied efficacies can shift the conformational equilibrium of a GPCR towards different conformations of the receptor, which in turn can activate one or another intracellular protein. Although several spectroscopy studies (e.g., for the β2-adrenergic receptor, herein referred to as B2AR, see [7]–[9]) have been instrumental in showing that ligands with different efficacies stabilize GPCR conformational states that are structurally and kinetically distinguishable, perhaps the most direct evidence of ligand-induced conformational specificity comes from the recent high-resolution crystallographic structures of several different ligand-bound GPCRs. In the majority of cases, these structures were obtained in the presence of an inverse agonist, and therefore in an inactive state. Only very recently have high-resolution crystal structures of agonist-bound GPCRs started to appear in the literature [10]–[15]. However, possibly restrained by crystallization conditions, not all these agonist-bound structures present the features that are usually attributed to an active GPCR conformation, most typically: the large outward movement of transmembrane helix 6 (TM6) with respect to the center of the receptor helical bundle, which is accompanied by the disruption of an important salt bridge between the conserved D/E3.49-R3.50 pair and E6.30, commonly referred to as the “ionic lock”. Residue numbering here and throughout the text follows the Ballesteros-Weinstein notation [16]. According to this notation, each residue is indicated by a two-number identifier N1.N2 where N1 is the number of the transmembrane helix, and N2 is the residue number on that helix relative to its most conserved position, which is designated N2 = 50. We direct the reader elsewhere (e.g., [17], [18]) for recent reviews of all the relevant structural changes that have been attributed by various biophysical techniques to active forms of GPCRs.

A different extent of structural rearrangement was noted at the binding site of high-resolution crystal structures of GPCRs depending on the type of ligand to which they were bound. For instance, only minor local structural changes were noted between the high-resolution crystal structures of the B2AR in the presence of inverse agonists such as carazolol [19], timolol [20], ICI-*118,551* [21], or a compound deriving from virtual screening [21] and the neutral antagonist alprenolol [21]. Slightly more pronounced differences were noted by comparing these inverse agonist/antagonist-bound binding pockets with those stabilized by full agonists (i.e., either the covalently-bound ligand FAUC50 [11] or BI-167107 [10]). Among them, the most notable differences were the hydrogen bonding contacts that only agonists formed with S5.42 and S5.46 on TM5. Similar interactions helped discriminate between inverse agonist-bound crystal structures of the β1-adrenergic receptor (B1AR) and structures obtained in the presence of full agonists (e.g., isoprenaline or carmoterol) [13]. Notably, only one of these two hydrogen bonds involving TM5, specifically the interaction with S5.42, was also present in structures stabilized by the partial agonists salbutamol or dobutamine, suggesting a distinguishable binding mode between full and partial agonist structures [13]. Analogous to the cases of the B1AR and B2AR, where specific residues (i.e., S5.46) are found to bind uniquely to agonists, key residues (S7.42 and H7.43) that bind agonists (either adenosine or NECA) but not antagonists (ZM241385) were revealed by the very recent crystal structures of a thermostabilized construct of the adenosine A2A receptor [15]. Unlike another recent crystal structure of this receptor stabilized by both T4-lysozyme and the conformationally selective agonist UK-432097 [12], these agonist-bound structures did not display changes at the cytoplasmic side that resemble those of an active state of a GPCR. In addition to the crystal structure of the adenosine A2A receptor bound to UK-432097 [12], these more marked changes at the cytoplasmic side have so far only been observed in the high-resolution crystal structures of opsin [22], [23], Meta II rhodopsin [14], and the nanobody-stabilized B2AR [10].

Despite these recent remarkable achievements in structural biology of GPCRs, the majority of pharmacologically relevant ligands of these receptors do not appear to be ideally suited for the stabilization and crystallization of these receptors, most likely because of their low affinity, slow off-rate, and poor solubility. Not only might this prevent the identification of physiologically relevant conformational states of a given GPCR, but it is considered a limiting bottleneck for the characterization of different structures of these receptors. Molecular dynamics (MD) simulations can help to fill this information gap by enabling an atomic-level characterization of ligand-specific conformations that are impossible or difficult to retrieve experimentally. Moreover, these simulations allow extension of static structural data into dynamic representations, thus laying the basis for a mechanistic understanding of the selective activation of GPCR-mediated signaling pathways.

To enable characterization of large conformational changes within the limited timescales commonly accessible to MD simulations, and to evaluate the extent to which ligands with different efficacies affect the free-energy landscape of GPCRs, we implemented a computational strategy employing a combination of different adaptive biasing techniques. Specifically, we used well-tempered metadynamics [24] to identify metastable states of a GPCR along putative activation pathways between inactive and active crystallographic states determined by adiabatic biased MD. We tested the accuracy of this strategy in reproducing crystallographic [19], [21] and/or spectroscopic [7]–[9] data available for the B2AR in its interaction with either a full agonist (i.e., epinephrine), a weak partial agonist (i.e., dopamine), a very weak partial agonist (i.e., catechol), two inverse agonists (i.e. ICI-*118,551* and carazolol), or one neutral antagonist (i.e., alprenolol). The results show a clear ligand-induced modulation of the free-energy landscape of the receptor with shifts in the conformational equilibrium towards inactive or active conformations depending on the physiological response elicited by the simulated ligand.

## Materials and Methods

### System and Simulation Setup

A model of the B2AR (Figure S1 was prepared starting from one of the available crystal structures of this receptor (PDB ID: 2RH1), removing the lysozyme insertion, and modeling the missing intracellular loop 3 (IL3) with the Rosetta *ab-initio* loop modeling protocol [25]. The intracellular loop 2 (IL2), which is probably misfolded [26], [27] in the inactive structure of the B2AR (2RH1), but in a helical conformation in the active nanobody-stabilized crystal (3P0G) of the receptor, was also replaced by the lowest-energy Rosetta model with a helical fold. The resulting receptor model was embedded into an explicit 1-palmitoyl-2-oleoyl-sn-glycero-3-phosphocholine (POPC)/10% cholesterol membrane bilayer using a pre-equilibrated 8×8×10 nm patch hydrated with SPC/E water molecules, and the procedure described in [28]. As found in the crystal structure [19], one palmitoyl group was covalently attached to a C-terminal residue (Cys 341) of the receptor before insertion in the membrane. The system was then hydrated with SPC/E water molecules [29] and Na^+^ and Cl^–^ ions were added to ensure charge neutrality.

The resulting system of ∼50,000 total atoms was equilibrated with unbiased MD simulations for 20 nanoseconds (ns) using the Optimized Potentials for Liquid Simulations all-atom (OPLS-AA) force field [30] for the receptor and united-atoms Berger parameters for the lipids [31]. The Gromacs 4.0.7 [32] package enhanced with the Plumed plug-in [33] was used for all simulations. Specifically, NPT simulations were carried out under periodic boundary conditions, using the Parrinello-Rahman algorithm [34] with a time constant of 1.0 ps and a reference pressure of 1 bar to control pressure, and the Nose-Hoover [35] algorithm with a time constant of 1.0 ps to maintain a constant temperature of 300 K. Prior to production runs (summarized in Table S1), the system was equilibrated by a series of three 0.2 ns runs with progressively weaker restraints on the protein backbone followed by a 3.0 ns unconstrained equilibration. We used the standard Gromacs leap-frog [32] algorithm with a time step of 2.0 fs, LINCS algorithm [36] to preserve the bond lengths, and SETTLE algorithm [37] to maintain the geometry of the water molecules. Lennard-Jones interactions were treated with a twin-range cutoff of 0.9∶1.4 nm and an integration time step of 2.0 fs; the neighbor list was updated every 10 steps. Electrostatic interactions were described using the particle-mesh Ewald method [38], with a cutoff of 0.9 nm for real-space interactions, and a 0.12-nm grid with fourth-order B-spline interpolation for reciprocal-space interactions.

### Adiabatic Biased MD Simulations

The starting equilibrated unliganded conformation of the B2AR within the lipid bilayer was subjected to ten independent Adiabatic Biased MD (ABMD) simulations [39], [40] to obtain transition pathways of the receptor from an inactive to an active conformation, built using the coordinates of the nanobody-stabilized crystal structure (PDB code: 3P0G). Briefly, this method biases the system towards a given value χ_0_ of a predefined order parameter χ(R), where R represents the coordinates of the atoms in the system. A harmonic bias acts only when the distance of χ(R) from the target χ_0_ is bigger than its minimum value previously reached during the simulation (i.e. if χ(R(t))- χ_0_ > min_s<t_ χ(R(s))- χ_0_), according to the following equation:
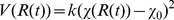
(1)


The order parameter χ measures the distance from the putative activated conformation of the receptor, and is defined as the Cα root mean square deviation (RMSD) from the active conformation of the B2AR (all residues were included except the long flexible IL3). In order to obtain activated final states, the simulation was run with χ_0_ = 0. After carrying out 10 independent ABMD runs with an elastic constant of k = 10 kcal/(mol⋅nm^2^), the trajectories were pooled and clustered using an average linkage agglomerative algorithm and the same dissimilarity measure used to run ABMD.

### Ligands Parameters

Bonded and van der Waals interactions for the ligands were assigned manually choosing the appropriate OPLS-AA atom types [30] for each atom in the molecule. Coulomb point charges were obtained according to the RESP approach [41] from quantum chemical calculations (i.e., geometry optimization using Gaussian 03 [42] and restricted Hartree-Fock calculations with the 6-31G* basis set).

### Ligand Docking

Ligands for which an experimental crystal structure in complex with the B2AR is available, i.e. 2RH1 for carazolol [19], 3NY8 for ICI-*118,551* [21], and 3NYA for alprenolol [21], were positioned in the binding pocket accordingly. The other ligands, i.e. the full agonist epinephrine and the partial agonists dopamine and catechol, were docked into the initial inactive model of B2AR, using a standard Autodock 4.0 protocol [43], [44]. Inferences from agonist-bound crystal structures of B2AR [10], [11] and B1AR [13] were taken into account when selecting the most accurate initial binding poses of these ligands for free-energy calculations. Notably, simulations of initial conformations comprising slightly different binding poses produced similar free-energy profiles (data not shown).

### Metadynamics

The free-energy profiles of liganded and unliganded systems were estimated using metadynamics [45]–[47]. Briefly, this technique enables an efficient reconstruction of the free-energy as a function of a set of *k* predetermined order parameters, referred to as collective variables s*_i_*(R), 1≤*i*≤*k*. A history-dependent bias potential is added to the force-field driving the system dynamics so as to discourage the re-visiting of regions of the s*_i_* phase space that have already been explored. Specifically, the bias potential is
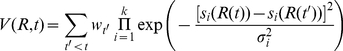
(2)where t′ is a multiple of a deposition time τ and the values of w_t′_ and σ_i_ regulate the shape and size of the Gaussian bias contributions. In the original metadynamics algorithm, w_t′_ = w is constant, and the free-energy profile can be estimated up to an insignificant additive time-dependent constant as W(R)  =  − lim_t→∞_ V(R,t).

Here, we used well-tempered metadynamics [24], a variant of the original metadynamics algorithm that enables assessment of simulation convergence while keeping the computational effort focused on physically relevant regions of the conformational space. In this variant of the method, the value of w_t′_ depends on the bias accumulated up to t′ according to the equation: 
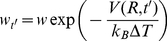
(3)where ΔT is a constant with the dimension of a temperature, *k*_B_ is the Boltzmann constant, and w is a constant energy representing the maximum height of the Gaussian biases. Since in the regions where the bias is higher the exponential factor reduces the rate of the bias update, the bias potential smoothly converges to a constant value in time, and the underlying free-energy can be derived by
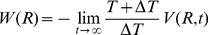
(4)where T is the temperature at which the simulation is performed.

To efficiently sample the conformational space along the activation pathway, reference states from the clustered ABMD runs were selected by cutting the agglomerative tree at 30 clusters, and selecting from them *n* = 10 clusters homogeneously covering the pathway. The reference states R_j_ (1≤*j*≤*n*) were numbered assigning j = 1 to the cluster closer to the inactive state (Cα RMSD from 2RH1 ∼0.4 Å) and j = 10 to the one closer to the active state (Cα RMSD from 3P0G ∼0.3 Å). Two path collective variables describing the position along (*s*) and the distance from (*z*) the pathway were defined [48] as follows:
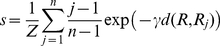
(5)

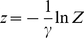
(6)where d(R,R_j_) is the squared Cα RMSD (excluding IL3) with respect to the reference structure R_j_, and Z  =  ∑_1≤*j≤n*_ exp(− γ d(R,R_j_)). The simulations were performed choosing γ  =  1/0.25 Å^-2^, σ_s_ = 0.1, and σ_z_ = 1 Å^2^, and well-tempered metadynamics was used with a bias factor ΔT = 10 T, an initial value of w = 0.4 kcal/mol, and a deposition interval τ = 8 ps. Metadynamics simulations were run for 300 ns, time at which the reconstructed free-energy difference between the metastable states converged to 0.2 kcal/mol.

### Free-Energy Calculations

Since the trajectory was generated adding the metadynamics bias, the resulting conformations cannot be used to obtain statistical information on order parameters other than the collective variables. However, it is possible to unbias the distribution of any given function of the system coordinates using the algorithm described in [49]. This so-called reweighting technique was used to estimate the free-energy surface of the complexes as a function of three important descriptors of receptor activation, namely the distance between R3.50 and E6.30 (the “ionic lock”), the rotamer of residue W6.48 (the so-called “toggle switch”), and the outward displacement of the intracellular segment of TM6.

Three order parameters were defined to monitor the behavior of these changes upon activation. For the ionic lock, we defined d_IL_ =  ||〈**R**_3.50_〉 − 〈**R**_6.30_〉||, where 〈**R**_3.50_〉 and 〈**R**_6.30_〉 represent the center of mass of the η-nitrogens of R3.50 and the δ-oxygens of E6.30, respectively. For the toggle switch, we monitored the first dihedral angle χ_TS_ of the side chain of W6.48. Finally, the movement of TM6 was measured by aligning the receptor to the inactive crystal structure (2RH1) and calculating the distance d_TM6_  =  ||**M** − 〈**R**_6.35_〉|| (angled brackets indicate the centroid of all the atoms of the residues) between the midpoint of an imaginary line connecting residues K6.35 and Y2.41 in the inactive structure, **M**  =  ½[〈**R**_2.41_〉 + 〈**R**_6.35_〉] (located roughly at the center of the intracellular exposed surface of the receptor), and residue K6.35. The outward movement is described by the difference in d_TM6_ values between any given conformation and the reference inactive crystal structure, i.e. by Δd_TM6_ = d_TM6_–d_TM6_ (2RH1).

### Unbiased Simulations

Representative conformational states of the metastable energy basins identified by metadynamics were selected and their structural stability analyzed. Specifically, standard, unbiased, NPT molecular dynamics simulations of these conformational states were initiated by randomizing new initial starting velocities with the Maxwell distributions at 300 K, and were run for ∼50 ns using the same simulation parameters described above.

## Results

We calculated the free-energy profile of the B2AR in an explicit POPC/10% cholesterol membrane bilayer along an activation pathway connecting two recently determined inactive [19] and active [10] crystallographic states of the receptor. Specifically, the receptor was studied in its unliganded form as well as in complex with the full agonist epinephrine, the weak partial agonist dopamine, the very partial agonist catechol, the inverse agonist ICI-*118-551*, the inverse agonist carazolol, or the neutral antagonist alprenolol. All free-energy values at the active and inactive states, and the barriers between them, are summarized in Table 1.

**Table 1:** Relative free−energy values (in kcal/mol) of the inactive, intermediate, and active states of the unliganded and liganded B2AR, together with the height of the barriers separating them.

	Inactive (s∼0.2)	TS_1_	Intermediate (s∼0.6)	TS_2_	Active (s∼0.9)
Unliganded	0.0^*^	3.0	0.0	−	−
Alprenolol	0.0^*^	3.3	1.0	−	−
Carazolol	0.0^*^	5.9	4.0	−	−
ICI-*118,551*	0.0^*^	5.2	3.8	−	−
Epinephrine	1.0	4.1	1.8	6.0	0.0^*^
Catechol	1.0	2.2	0.0^*^	−	−
Dopamine	1.5	4.2	0.0^*^	−	−

TS_1_ represents the transition state between the inactive and the intermediate states, while TS_2_ represents the transition state between the intermediate state and the active one. The most stable state for each system is indicated with a star.

### Unliganded Receptor

**Figure 1:**
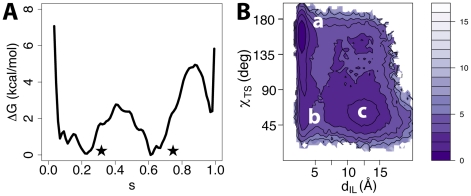
Free-energy of the unliganded receptor. (A) Free-energy as a function of the position (*s*) along the activation pathway. Note that the curve has been shifted so that the lowest energy minima (indicated by stars) correspond to a reference free-energy value. (B) Free-energy as a function of ionic lock distance (d_IL_) and the toggle switch dihedral (χ_TS_) molecular switches for the unliganded receptor; contour lines are reported every k_B_T.

An activation pathway from the inactive to the active B2AR crystal structures (PDB codes 2RH1 and 3P0G, respectively) was obtained by ABMD following the protocol described in the Materials and Methods section. This pathway was used to define the *s* and *z* collective variables (see the Materials and Methods section for corresponding equations) that were employed for the metadynamics simulations. Panel A of Figure 1 illustrates the free-energy ?G of the unliganded receptor as a function of the position *s* along the activation pathway following integration of the dependence on *z*. Specifically, *s* = 0.0 and *s* = 1.0 indicate the inactive and fully activated extreme conformations of the pathway, respectively. This free-energy profile shows two minima, one at s∼0.2 that is close to the inactive state and the other at s∼0.6 that is shifted towards the active state. The two states are separated by a barrier of ∼2.5 kcal/mol, but they have a similar overall stability (ΔG<k_B_T), and are therefore equally populated at equilibrium. Inspection of the entire two-dimensional free-energy profile ΔG(*s,z*) reported in the supplementary material (see panel A of Figure S2) shows that these states correspond to conformations along the activation pathway with values of *z* close to 0. Visual inspection of a representative structure of the s∼0.2 energy basin confirms that the corresponding transmembrane bundle is very close to the inactive B2AR crystal structure (Cα RMSD excluding IL3 ∼0.6 Å), as substantiated by the very small outward movement of TM6 (Δd_TM6_ ∼ 0.4 Å) with respect to the inactive crystal (see panel B of Figure S2). In contrast, a representative structure of the second energy basin at s∼0.6 (RMSD ∼1.6 Å and ∼1.1 Å from the inactive and active crystal structures, respectively) displays a more pronounced outward movement of TM6 (Δd_TM6_ ∼2.5 Å in Figure S2).

Figure 1B shows the free-energy of the unliganded B2AR as a function of order parameters that monitor molecular switches which have traditionally been reported as descriptors of GPCR activation. Specifically, these molecular switches are: 1) the ionic lock between TM3 and TM6, herein monitored using the distance *d_IL_* between R3.50 and E6.30 and 2) the W6.48 rotamer toggle switch, herein monitored using the first dihedral angle χ_TS_ of the residue side chain. Whilst the latter has not been observed in recent activated crystal structures of GPCRs, compelling spectroscopic data exist supporting a rotamer change of the W6.48 side chain upon activation [50]. Two different energy basins can be identified in the plot of Figure 1B: a more stable one, labeled *a*, in which both molecular switches are in their inactive conformation (d_IL_∼3 Å and χ_TS_∼163°), and a second basin, labeled *c*, where both switches are in their activated conformation (d_IL_∼12 Å and χ_TS_∼55°). The two basins are separated by a barrier of ∼3.0 kcal/mol. A transition state at χ_TS_∼65° and d_IL_∼5 Å (labeled *b* on the free-energy map) suggests a preferential rotamer toggle switch prior disruption of the ionic lock.

### Receptor Bound to a Neutral Antagonist

**Figure 2:**
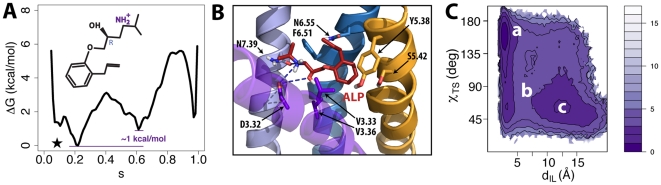
Simulation results for B2AR bound to the neutral antagonist alprenolol. (A) Free-energy profile as a function of the position (*s*) along the activation pathway. Note that the curve has been shifted so that the minimum (indicated by a star) corresponds to a reference free-energy value. (B) Binding mode of alprenolol. Relevant residues interacting with the ligand (any atom within a 3 Å distance cutoff) are indicated in stick representations. Helices TM5, TM6 and TM7 are shown in orange, blue and light blue respectively. Helix TM3 is shown in purple transparent representation whereas TM4 has been removed for clarity. (C) Free-energy as a function of ionic lock distance (d_IL_) and the toggle switch dihedral (χ_TS_) molecular switches.

The neutral antagonist alprenolol, consisting of an “aromatic head” (a 2-allyl-pheniloxyl moiety) and an “aliphatic tail” (oxy-propanol-amine) (see chemical structure in Figure 2A), was docked in accordance to the binding mode assumed by the ligand in the crystal structure of the corresponding ligand-bound receptor [21]. The results of the simulations for the alprenolol-bound receptor are illustrated in panels A-C of Figure 2. As shown in Figure 2A, the overall shape of the free-energy profile of the alprenolol-bound B2AR as a function of the position (*s*) along the activation pathway is qualitatively similar to the profile obtained for the unliganded receptor, and reported in Figure 1A. A similarity is also noted between the two-dimensional energy surfaces of the alprenolol-bound (Figure S3A) and the unliganded B2AR (Figure S2A). In spite of these qualitative similarities, the inactive state at s∼0.2 is more stable (∼1 kcal/mol) than the intermediate state at s∼0.6 for the alprenolol-bound receptor compared to the unliganded one. Given the relatively higher stability of the alprenolol-bound receptor conformation with no significant outward movement of TM6 (Δd_TM6_ ∼0.4 Å at s∼0.2 in Figure S3B), these results suggest an energy profile that is more suitable for a very weak inverse agonist rather than a neutral antagonist. Notably, data are available in the literature in support of an inverse agonist [51], [52] (or even a partial agonist [53]) role for alprenolol.

Figure 2B shows a representative conformation of the lowest energy basin identified for the alprenolol-bound receptor. In this conformation, and similar to the corresponding crystal structure [21], the alprenolol charged moiety in its aliphatic tail forms interactions with polar residues D3.32 and N7.39, while the ligand aromatic head interacts with residues V3.33, V3.36, F6.51, N6.55, Y5.38, and S5.42, which define a cleft formed by TM3, TM5 and TM6. Figure 2C shows that the energetically most stable alprenolol-bound inactive state is characterized by inactive molecular switches (χ_TS_∼160° and d_IL_∼5 Å). This state, labeled *a* in Figure 2C, is separated by an energy barrier of ∼3 kcal/mol from the second most stable energetic minimum at χ_TS_∼50° and d_IL_∼12 Å (*c* in Figure 2C), with a transition state (*b* in Figure 2C) at χ_TS_∼85° and d_IL_∼5 Å. Thus, the presence of alprenolol in the binding pocket does not appear to disrupt the free-energy profile seen in the unliganded receptor, further confirming a possible rotamer toggle switch of the W6.48 residue prior breaking of the ionic lock. The stability of alprenolol in a representative state of the ligand-receptor complex extracted from the most stable energy basin at s∼0.2 was confirmed by carrying out ∼50 ns unbiased MD simulations. The evolution of the ligand and the protein RMSD during these simulations is reported in Figure S4.

### Receptor Bound to Inverse Agonists

**Figure 3:**
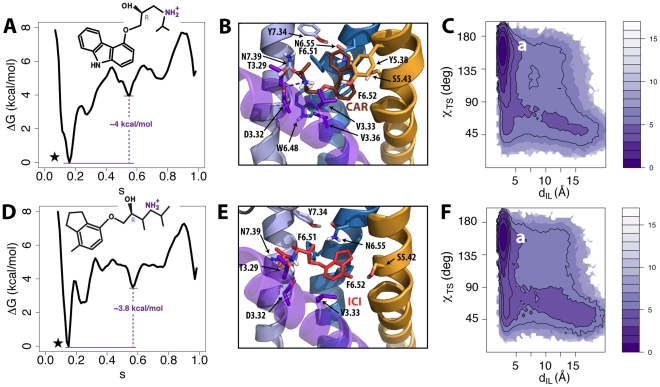
Simulation results for B2AR bound to the inverse agonists carazolol and ICI-*118,551*. (A and D) Free-energy profiles as a function of the position (*s*) along the activation pathway for carazolol and ICI-*118,551*, respectively. Note that the curves have been shifted so that the minima (indicated by stars) correspond to reference free-energy values. (B and E) Binding modes of carazolol and ICI-*118,551*, respectively. Relevant residues interacting with the ligands (any atom within a 3 Å distance cutoff) are indicated in stick representations. Helices TM5, TM6 and TM7 are shown in orange, blue and light blue respectively. Helix TM3 is shown in purple transparent representation whereas TM4 has been removed for clarity. (C and F) Free-energies as a function of ionic lock distance (d_IL_) and the toggle switch dihedral (χ_TS_) molecular switches for the carazolol- and ICI-*118,551*-bound B2AR, respectively.

We assessed the effect of two different B2AR inverse agonists, namely ICI-*118,551* and carazolol, on the free-energy landscape of the receptor during transition from inactive to activated experimental states. Carazolol and ICI-*118,551* share important structural features with alprenolol, e.g., they both have an “aliphatic tail” (oxy-propanol-amine for carazolol and oxy-butanol-amine for ICI-*118,551*) and an “aromatic head”. The results of the simulations for the carazolol-bound and the ICI-*118,551*-bound receptor are illustrated in panels A-C and D-F of Figure 3, respectively.

In the presence of either carazolol or ICI-*118,551*, the B2AR free-energy profiles (Figure 3A and 3D, respectively) show a single lowest-energy basin at s∼0.18 close to the inactive state of the receptor. These much more stable energy basins are also present in the two-dimensional energy surfaces of the carazolol-bound (Figure S5A) and the ICI-*118,551-*bound (Figure S5C) B2AR, and comprise inactive conformations as further illustrated by the lower energy values for states characterized by the absence of outward movement of TM6 (Δd_TM6_ ∼ 0.4 Å in Figures S5B and S5D). Representative conformations extracted from the lowest energy basins of either the carazolol-bound (Figure 3B) or the ICI-*118,551*-bound (Figure 3E) receptors show that the energy-optimized binding poses of these ligands are very similar to their positions in the corresponding crystal structures [19], [21]. Similar to the binding mode of alprenolol, the charged moieties contained in the aliphatic tails of these ligands interact with polar residues D3.32, and N7.39, while their aromatic heads are oriented toward TM3, TM5, and TM6, thus directly interacting with residues in these helices (see Figures 3B and 3E). To assess the stability of the ligands in these representative conformations, we performed standard, unbiased MD simulations. As shown in Figures S6A-D, which report the time evolutions of the RMSD of the protein, as well as those of the heavy atoms of carazolol and ICI-*118,551*, after superposition of the receptor Cα atoms, the receptor conformations are stable and the binding modes of the ligands are conserved over a simulation time of ∼50 ns.

The intermediate state at *s*∼0.6 that was significantly populated in the unliganded and neutral antagonist-bound receptor is much less stable at ΔG∼4.0 kcal/mol in the case of the carazolol-bound or ICI-*118,551*-bound receptors (see Figures 3A and 3D, respectively). However, these are still metastable states, as judged by the presence of shallow minima at s∼0.6 in both the free-energy profiles, and are separated from the inactive states by multiple barriers. In terms of modulation of the toggle switch and the ionic lock, the free-energy as a function of χ_TS_ and d_IL_ (Figures 3C and 3F for the carazolol-bound and ICI-*118,551*-bound complexes, respectively) features only one minimum in the inactive region of these molecular switches (χ_TS_∼160° and d_IL_∼3 Å).

### Receptor Bound to Agonists

**Figure 4:**
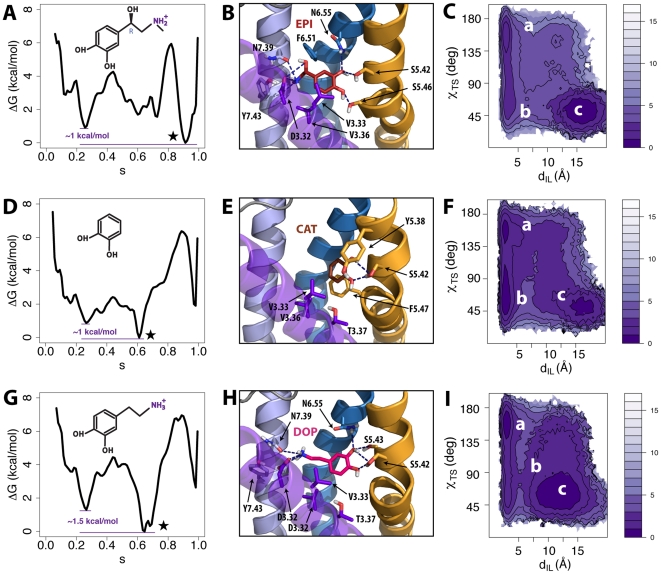
Simulation results for B2AR bound to the full agonist epinephrine, the very weak partial agonist catechol, and the weak partial agonist dopamine. (A, D, and G) Free-energy profiles as a function of the position (*s*) along the activation pathway for epinephrine, catechol, and dopamine, respectively. Note that the curves have been shifted so that the minima (indicated by stars) correspond to reference free-energy values. (B, E, and H) Binding modes of epinephrine, catechol, and dopamine, respectively. Relevant residues interacting with the ligands (any atom within a 3 Å distance cutoff) are indicated in stick representations. Helices TM5, TM6 and TM7 are shown in orange, blue and light blue respectively. Helix TM3 is shown in purple transparent representation whereas TM4 has been removed for clarity. (C, F, and I) Free-energies as a function of ionic lock distance (d_IL_) and the toggle switch dihedral (χ_TS_) molecular switches for the epinephrine-, catechol-, and dopamine-bound B2AR, respectively.

To study the effects of full agonists on the free-energy landscape of B2AR, we docked epinephrine into the receptor, and performed metadynamics calculations. Figure 4A shows the free-energy profile of the epinephrine-bound B2AR with the lowest energy state (s∼0.9) likely to correspond to an activated conformation. The same observation is possible by inspection of the two-dimensional free-energy surface (Figure S7A) as well as the TM6 outward movement (Δd_TM6_ ∼5.9 Å in Figure S7B) as a function of the position s along the activation pathway. However, a second low-energy metastable state is present in these free-energy profiles, close to the inactive state (s∼0.2), and with a free-energy difference of only ∼1 kcal/mol with respect to the most stable activated state.

As illustrated in Figure 4B, our proposed binding mode of epinephrine within a fully activated B2AR (energy basin at s∼0.9) is consistent with the binding poses displayed by full agonists in the B2AR [10] and B1AR [13] crystallographic structures. Specifically, the ligand amino group forms hydrogen bonds with D3.32 and N7.39 of B2AR, the ligand β-hydroxyl group interacts with D3.32, and the ligand catecholamine hydroxyl groups interact through hydrogen bonding with the side chains of both S5.42 and S5.46. In this state, the B2AR helix bundle is structurally very similar to the corresponding nanobody-activated crystal structure of the receptor (C? RMSD from 3P0G is ∼1.6 Å). The stability of the epinephrine binding pose and the specific receptor conformation were verified by carrying out ∼50 ns standard MD simulations (see corresponding time evolutions of RMSD in Figure S8). On the other hand, representative structures of the energy basin at s∼0.2 (data not shown) corresponded to conformations of the helix bundle very similar to the inactive crystal structure of B2AR (Cα RMSD from 2RH1 is ∼1.0 Å).

Two energy basins (labeled *a* and *c*) were identified from the free-energy as a function of the order parameters describing the ionic lock and rotamer toggle switches (Figure 4C). Specifically, the basin comprising conformations in which both the ionic lock and rotamer toggle switches are in the ‘active’ (d_IL_∼16 Å and χ_TS_∼50°) positions appear to be more stable than the basin with receptor conformations with ‘inactive’ (d_IL_∼3 Å and χ_TS_∼160°) molecular switches. Also in this case, the minimum free-energy path between these two energy basins suggests activation of the toggle switch prior breaking of the ionic lock interaction along the path to full receptor activation.

### Receptor Bound to Partial Agonists

**Figure 5:**
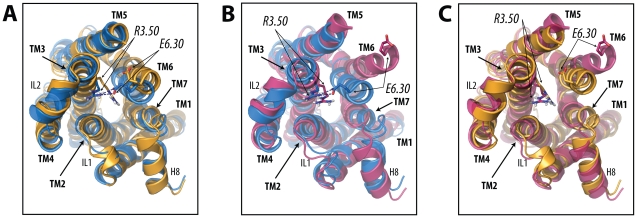
Structural comparisons between ligand-specific B2AR conformations. Specifically, these comparisons (viewed from the intracellular side) are between: (A) an inverse agonist (carazolol)-bound inactive state at s∼0.2 (blue color) and a partial agonist (dopamine)-stabilized intermediate conformation at s∼0.6 (orange color); (B) an inverse agonist (carazolol)-bound inactive state at s∼0.2 (blue color) and a full agonist (epinephrine)-stabilized active conformation at s∼0.9 (pink color); and (C) a partial agonist (dopamine)-bound intermediate conformation at s∼0.6 (orange color) and a full agonist (epinephrine)-stabilized active conformation at s∼0.9 (pink color). The position of the side chains of the residues involved in the ionic locks are indicated with sticks. For clarity, IL3 has been removed.

The weak and very weak partial agonists, dopamine and catechol, were also simulated in the context of the B2AR activation pathway. Figures 4D and 4G illustrate the free-energy profiles of the catechol-bound and dopamine-bound receptors, respectively. In both cases the receptor is most stabilized in an intermediate state (s∼0.6) along the pathway to activation. Inspection of the free-energy as a function of the position (*s*) along and the distance (*z*) from the activation pathway (see Figures S9A and S9C for the catechol-bound and dopamine-bound receptors, respectively) confirms that these two ligands stabilize a state different from the inactive or fully activated ones as judged by the lowest energy values at z∼2 in Figure S9A for catechol, and at s∼0.6, z∼0.0 in Figure S9C for dopamine. This difference is also evident from the free-energy surfaces as a function of the TM6 outward movement and the position along the activation pathway (see Figures S9B and S9D, respectively), as well as from the structural superpositions shown in Figure 5. Specifically, Figure 5 illustrates the structural differences between the TM regions of the predicted inverse agonist- and partial agonist-specific conformations (Figure 5A), the inverse agonist- and full agonist-specific conformations (Figure 5B), and the partial agonist- and full agonist-specific conformations (Figure 5C).

Figures 4E and 4H show the binding modes of catechol and dopamine, respectively. These binding poses were proven to be stable during ∼50 ns of unconstrained MD simulations (see Figures S10A and S10B for the time evolution of the RMSD of catechol and dopamine, respectively, and Figures S10C and S10D for the time evolution of the RMSD of the corresponding protein Cα atoms). In agreement with inferences from recent B1AR structures co-crystallized with either full or partial agonists, these two B2AR partial agonists formed stable hydrogen bonds (through the catechol moiety) with the side chain of S5.42, but do not with S5.46. In terms of the ligand-induced modulation of the molecular switches, the catechol-bound B2AR state with a broken ionic lock (located at χ_TS_∼50° and d_IL_∼16 Å in Figure 4F) is relatively less stable than the corresponding larger basin identified in the presence of dopamine (see Figure 4I), consistent with spectroscopy data suggesting that catechol is unable to disrupt the ionic lock [9].

## Discussion

Understanding the molecular mechanisms underlying GPCR functional selectivity is extremely important in modern drug discovery, since it provides a unique opportunity for the identification or rational design of ‘biased’ ligands as novel more effective therapeutics. Epitomizing an emerging paradigm in current drug discovery [54], native states of GPCRs can be assumed in a dynamic equilibrium between different conformational sub-states [11], [18], [55], which correspond to the valleys of an energy landscape, the barriers of which reflect the timescales of the conformational exchange. The relative populations of these sub-states follow statistical thermodynamics distributions and are shifted towards specific conformations as a consequence of ligand binding and/or other allosteric events such as those induced by protein-protein interactions. Thus, ligands with varied efficacies are believed to modulate the free-energy landscape of a GPCR, shifting the conformational equilibrium towards active or inactive conformations of the receptor, depending on their pharmacological action.

A reliable characterization of the specific conformations that inverse agonists, agonists (both full and partial), or antagonists can stabilize in a given GPCR is highly desirable for the structure-based discovery of novel ligands eliciting selected functional responses. This is difficult to achieve by X-ray crystallography for the majority of GPCRs due to their intrinsic structural instability, and the realization that the majority of pharmacologically active ligands are not ideal compounds for receptor stabilization that is suitable for crystallization.

The enhanced sampling approach we describe here provides atomic-resolution information of receptor conformations along pre-determined activation pathways that are differentially stabilized by ligands with different efficacies. Our approach also provides a quantitative description of the thermodynamics of the B2AR basal activity, with the unliganded receptor being able to sample both an inactive state and an intermediate state that is shifted towards the activated conformation. This latter state is structurally different from the fully active state of B2AR captured by the nanobody-stabilized crystal structure. Although it exhibits a broken ionic lock and a cytoplasmic opening that is able to accommodate the camelid antibody, a few clashes are produced by the much smaller outward movement of TM6 (∼2.5 Å compared to the ∼5.9 Å that can be achieved by a full agonist). Given the small free-energy difference between the two lowest energy minima identified for the unliganded B2AR, these two states are almost equally populated at equilibrium, in agreement with the high basal activity of the B2AR. Moreover, the relatively low energy barrier between the two states is consistent with the flexible nature of the unliganded B2AR, and the consequent difficulty in obtaining crystals of the native receptor.

We observed a more or less pronounced perturbation of the free-energy profile of the unliganded B2AR depending on the ligand considered for binding. Although alprenolol has often been described as a neutral antagonist of B2AR, its presence in the B2AR binding pocket slightly modifies the free-energy profile of the receptor, making the inactive state more stable in spite of the small difference in free-energy (∼k_B_T). This result is not completely surprising in light of the evidence existing in the literature for a role of alprenolol as an inverse agonist or even a weak agonist, depending on the assay used 56,57.

Our results show that the selection of a single conformational state is particularly effective in the case of inverse agonists. The docking of either carazolol or ICI-*118,551* in the receptor dramatically changes the free-energy landscape of B2AR and reduces it to a funneled profile with a single major basin corresponding to the inactive conformation. This result is consistent with the greater availability of crystals of B2AR in an inactive conformation stabilized by potent inverse agonists in the binding pocket, and with the observation that the structural features of the inactive states of the various receptors obtained so far are similar.

The situation is different when we study the free-energy landscape in the presence of agonists. The computational experiment with epinephrine shows that a full agonist is capable of stabilizing a state of B2AR presenting structural features that have been found in the nanobody-stabilized agonist-bound crystal structure of B2AR. However, in addition to this active state, we obtain a relatively stable agonist-bound inactive state that is structurally similar to the inverse agonist-bound crystal structure of B2AR. This is not surprising, given the absence of TM6 outward movements noted in both the B2AR crystal structure with a covalently-bound agonist [11], and the agonist-bound B1AR crystal structures [13]. Moreover, the relatively small difference in free-energy between the fully active and the inactive agonist-bound conformations is probably due to the lack of the G-protein in the simulation setup, in line with the observation deriving from the two recent agonist-bound B2AR crystal structures [10], [11] that a ligand alone is not sufficient to stabilize a fully active crystallographic state of the receptor, but a G-protein mimicking nanobody is necessary to trap this conformation. Different from the crystallographic information, but in line with experimental evidence from fluorescence spectroscopy [9], we find that metastable states corresponding to fully (and partial) activated conformations of the receptor favor the rotamer change of the W6.48 side chain.

The partial agonism elicited by dopamine and catechol shifts the conformational equilibrium towards states that are different from that stabilized by the full agonist, and captured in the nanobody-stabilized crystal structure. In particular, the two ligands affect the free-energy landscape in different ways. While the intermediate dopamine-bound state always features a broken ionic lock, the receptor samples conformations that have different ionic lock states when catechol is in the binding pocket. Notably, experimental evidence from fluorescence spectroscopy [9] also suggested that the very weak partial agonist catechol is not able to completely disrupt the interaction between the charged residues at the cytoplasmic end of TM3 and TM6. Structurally, the two conformations stabilized by catechol and dopamine are different in the degree of separation between the extracellular ends of TM5 and TM6 and between the intercellular ends of TM3 and TM6. Consistent with the hypothesis that global structural features of the receptor, such as the tilt of the extracellular half of TM5, can optimize the binding to agonists [58], we see a larger TM5 tilt in the presence of dopamine (as well as for epinephrine) and a smaller one in the presence of catechol. Owing to the greater ability of catechol to stabilize a state with a formed ionic lock, the intracellular ends of TM3 and TM6 also appear slightly closer (by ∼1 Å) together.

In summary, we have designed a strategy using a combination of different adaptive biasing techniques that enables characterization of reliable ligand-specific conformations as demonstrated here in the case of B2AR. The strategy is completely general and may be of practical use for the structure-based design of ‘biased’ ligands that selectively activate signaling pathways, and may therefore exhibit improved therapeutic properties.

## Supporting Information

Figure S1 **Ribbon representation of the B2AR illustrating the secondary structure motifs and the residues used to monitor activation.** Residues involved in the “ionic lock” (R3.50 and E6.30) and the “toggle switch” (W6.48) are indicated with sticks. The midpoint between residue Y2.41 and E6.30 is indicated with a purple dot and the approximate location of the binding pocket with a red sphere. Transmembrane helices are colored (TM1 in red, TM2 in orange, TM3 in purple, TM4 in brown, TM5 in yellow, TM6 in transparent blue, and TM7 in light blue).
(TIF)

Figure S2 **Additional analyses of the simulations of the unliganded B2AR.** (A) Free-energy of the unliganded B2AR as a function of the position along (*s*) and the distance from (*z*) the activation pathway. The surface has been shifted so that the lowest energy minima correspond to reference free-energy values; contours are spaced by 2 kcal/mol. (B) Free-energy projection as a function of the path variable *s* and the displacement of TM6. The latter is defined by the distance between the midpoint of an imaginary line connecting residues K6.35 and Y2.41 (roughly at the center of the intracellular exposed surface of the receptor) and residue K6.35.
(TIF)

Figure S3 **Additional analyses of the simulations of the B2AR bound to the neutral antagonist alprenolol.** (A) Free-energy of the as a function of the position along (*s*) and the distance from (*z*) the activation pathway. The surface has been shifted so that the lowest energy minima correspond to reference free-energy values; contours are spaced by 2 kcal/mol. (B) Free-energy projection as a function of the path variable *s* and the displacement of TM6. The latter is defined by the distance between the midpoint of an imaginary line connecting residues K6.35 and Y2.41 (roughly at the center of the intracellular exposed surface of the receptor) and residue K6.35.
(TIF)

Figure S4 **Time evolution of the RMSD of alprenolol and B2AR.** RMSD vs. time of (A) the alprenolol heavy atoms after alignment of the B2AR Cα atoms and (B) the B2AR Cα atoms with respect to the initial conformation. The initial structure was extracted from the s∼0.2 and z∼0.0 Å basin in figure S3.
(TIF)

Figure S5 **Additional analyses of the simulations of the B2AR bound to the inverse antagonists carazolol and ICI-** ***118,551*** **.** (A and C) Free-energy of carazolol- and ICI-*118,551*-bound B2AR as a function of the position along (*s*) and the distance from (*z*) the activation pathway. The surface has been shifted so that the lowest energy minima correspond to reference free-energy values; contours are spaced by 2 kcal/mol. (B and D) Free-energy projection as a function of the path variable *s* and the displacement of TM6 for the inverse agonists carazolol- and ICI-*118,551*-bound B2AR, respectively. The latter is defined by the distance between the midpoint of an imaginary line connecting residues K6.35 and Y2.41 (roughly at the center of the intracellular exposed surface of the receptor) and residue K6.35.
(TIF)

Figure S6 **Time evolution of the RMSD of carazolol, ICI-** ***118,551*** **, and B2AR.** RMSD vs. time of the (A) carazolol and (B) ICI-*118,551* heavy atoms after alignment of the B2AR Cα atoms, and the B2AR Cα atoms in the simulations with (C) carazolol or (D) ICI-*118,551* with respect to the initial structure. The initial structure was extracted from the s∼0.2 and z∼0.0 Å basins in Figure S5.
(TIF)

Figure S7 **Additional analyses of the simulations of the B2AR bound to the full antagonist epinephrine.** (A) Free-energy of the full agonist epinephrine-bound B2AR as a function of the position along (*s*) and the distance from (*z*) the activation pathway. The surface has been shifted so that the lowest energy minima correspond to reference free-energy values; contours are spaced by 2 kcal/mol. (B) Free-energy projection as a function of the path variable *s* and the displacement of TM6. The latter is defined by the distance between the midpoint of an imaginary line connecting residues K6.35 and Y2.41 (roughly at the center of the intracellular exposed surface of the receptor) and residue K6.35.
(TIF)

Figure S8 **Time evolution of the RMSD of epinephrine and B2AR.** RMSD vs. time of (A) the epinephrine heavy atoms after alignment of the B2AR Cα atoms, and (B) the B2AR Cα atoms with respect to the initial structure. The initial structure was extracted from the s∼0.9 and z∼0.5 Å basins in Figure S7.
(TIF)

Figure S9 **Additional analyses of the simulations of the B2AR bound to the partial agonists catechol and dopamine.** (A and C) Free-energy of the partial agonists catechol- and dopamine-bound B2AR as a function of the position along (*s*) and the distance from (*z*) the activation pathway. The surface has been shifted so that the lowest energy minima correspond to reference free-energy values; contours are spaced by 2 kcal/mol. (B and D) Free-energy projection as a function of the path variable *s* and the displacement of TM6 for the partial agonists catechol- and dopamine-bound B2AR, respectively. The latter is defined by the distance between the midpoint of an imaginary line connecting residues K6.35 and Y2.41 (roughly at the center of the intracellular exposed surface of the receptor) and residue K6.35.
(TIF)

Figure S10 **Time evolution of the RMSD of catechol, dopamine, and B2AR.** RMSD vs. time of the (A) catechol and (B) dopamine heavy atoms after alignment of the B2AR Cα atoms, and of the B2AR Cα atoms in the simulations with (C) catechol or (D) dopamine with respect to the initial structure. The initial structure was extracted from the s∼0.6 basins in Figure S9.
(TIF)

Table S1 **List of the production runs performed in this study.** Number and length of independent simulations carried out for each system.
(DOC)
