# Supplementary figures and images for: Ligand-Induced Modulation of the Free-Energy Landscape of G Protein-Coupled Receptors Explored by Adaptive Biasing Techniques
Source: PLoS Comput Biol. 2011 Oct 13;7(10):e1002193. doi: 10.1371/journal.pcbi.1002193 (PMC3192824; doi:10.1371/journal.pcbi.1002193)

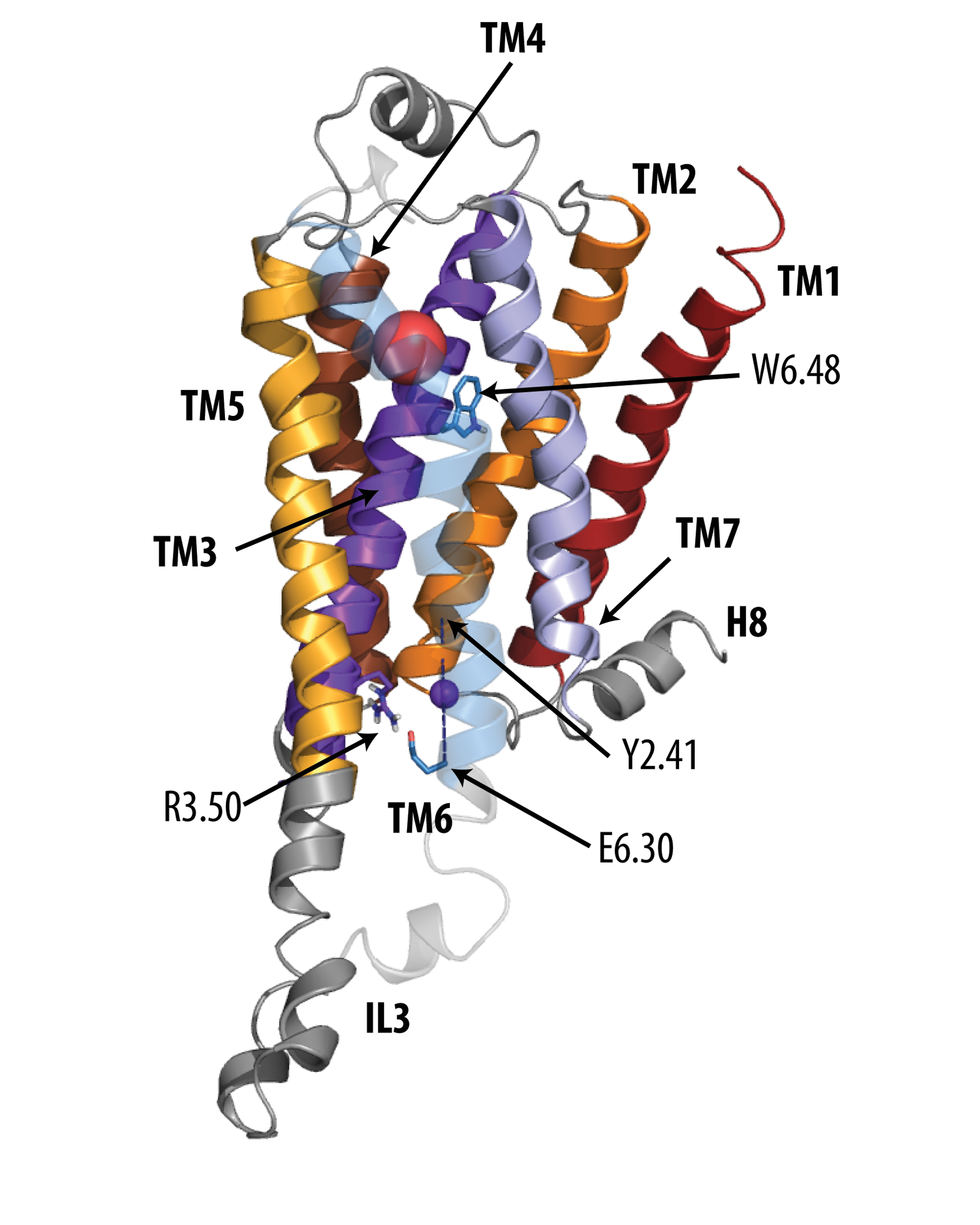

Supplement: Figure S1 — Ribbon representation of the B2AR illustrating the secondary structure motifs and the residues used to monitor activation. Residues involved in the “ionic lock” (R3.50 and E6.30) and the “toggle switch” (W6.48) are indicated with sticks. The midpoint between residue Y2.41 and E6.30 is indicated with a purple dot and the approximate location of the binding pocket with a red sphere. Transmembrane helices are colored (TM1 in red, TM2 in orange, TM3 in purple, TM4 in brown, TM5 in yellow, TM6 in transparent blue, and TM7 in light blue). (TIF) [file pcbi.1002193.s001.tif]

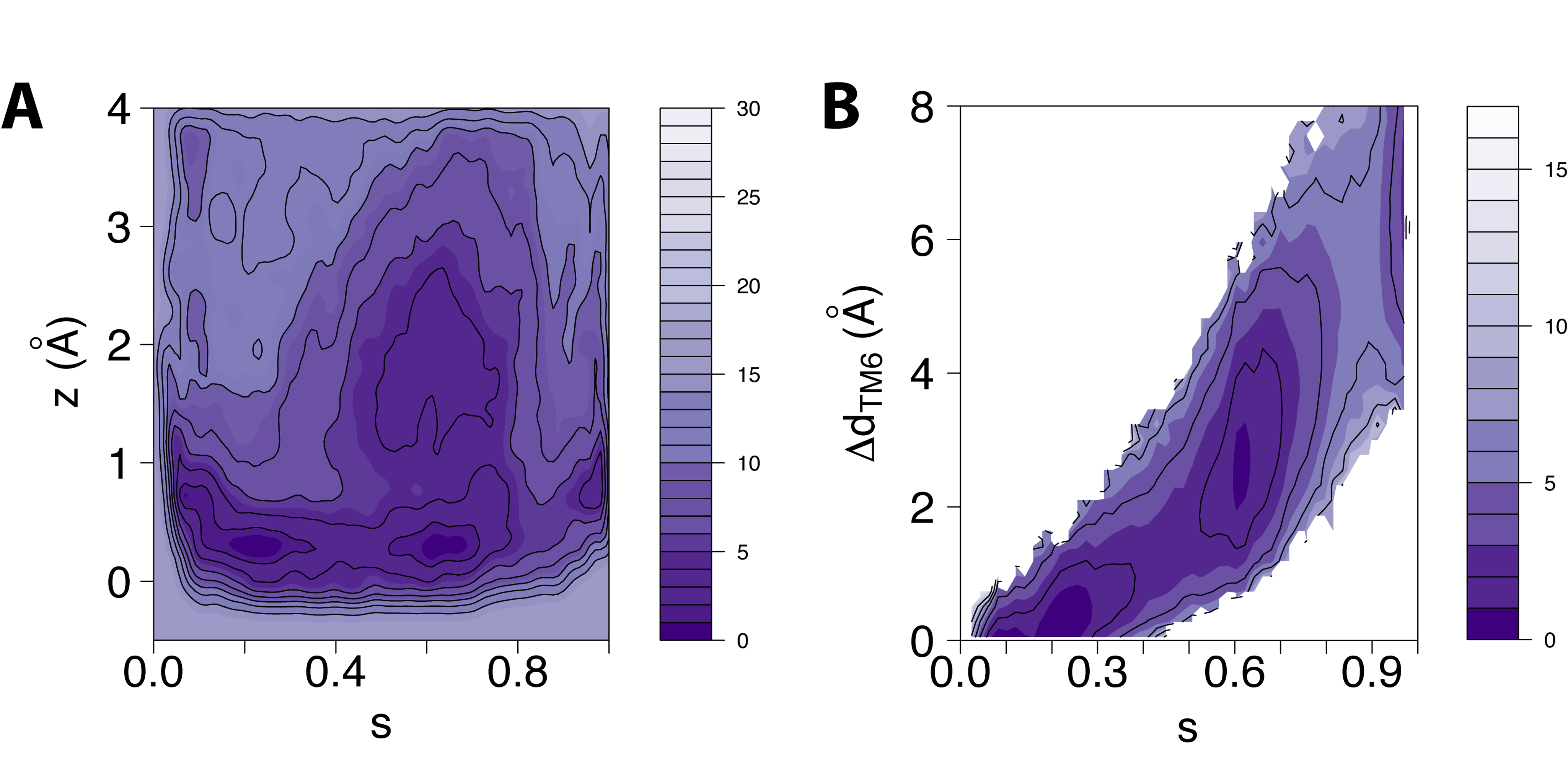

Supplement: Figure S2 — Additional analyses of the simulations of the unliganded B2AR. (A) Free-energy of the unliganded B2AR as a function of the position along (s) and the distance from (z) the activation pathway. The surface has been shifted so that the lowest energy minima correspond to reference free-energy values; contours are spaced by 2 kcal/mol. (B) Free-energy projection as a function of the path variable s and the displacement of TM6. The latter is defined by the distance between the midpoint of an imaginary line connecting residues K6.35 and Y2.41 (roughly at the center of the intracellular exposed surface of the receptor) and residue K6.35. (TIF) [file pcbi.1002193.s002.tif]

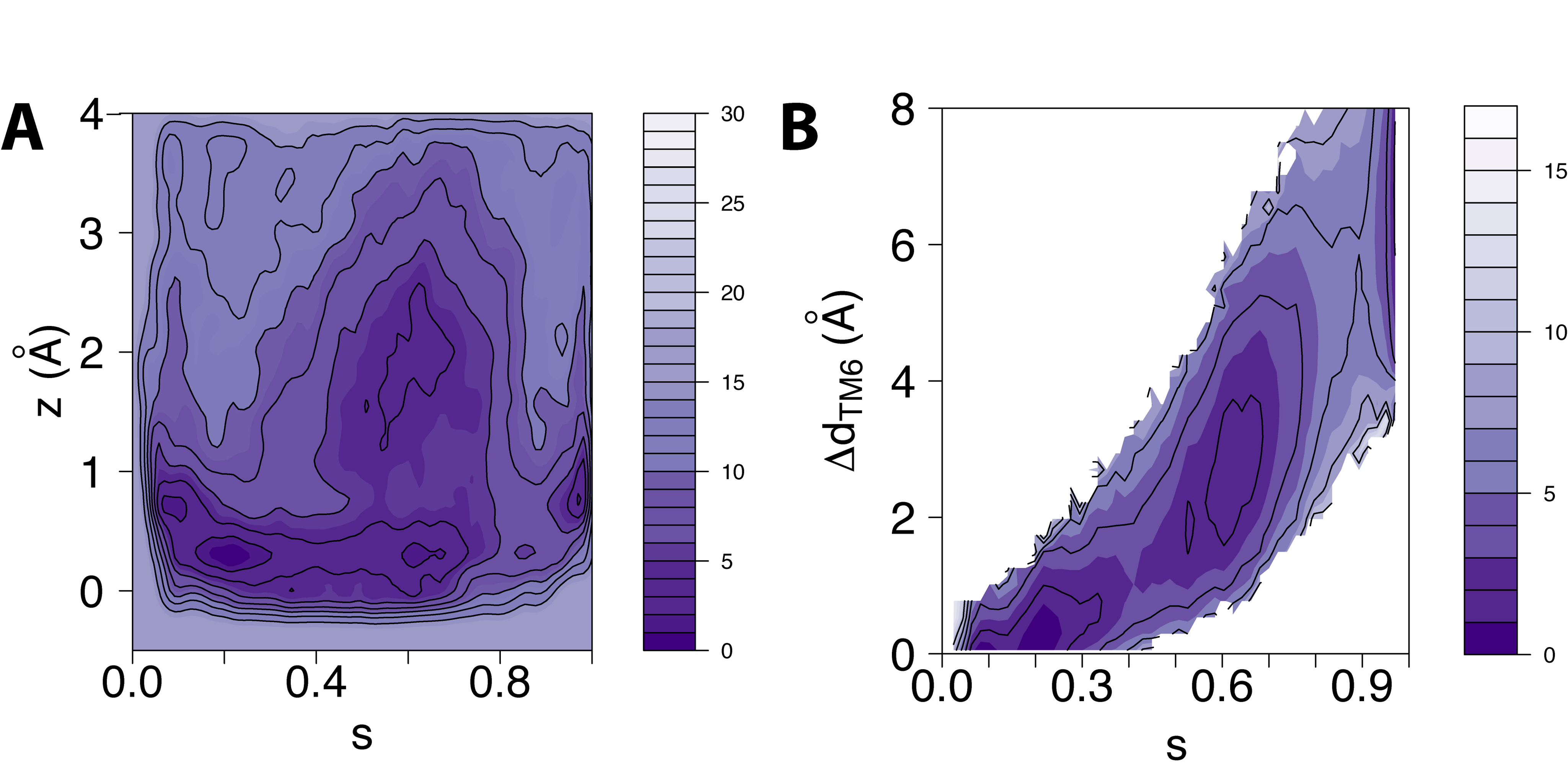

Supplement: Figure S3 — Additional analyses of the simulations of the B2AR bound to the neutral antagonist alprenolol. (A) Free-energy of the as a function of the position along (s) and the distance from (z) the activation pathway. The surface has been shifted so that the lowest energy minima correspond to reference free-energy values; contours are spaced by 2 kcal/mol. (B) Free-energy projection as a function of the path variable s and the displacement of TM6. The latter is defined by the distance between the midpoint of an imaginary line connecting residues K6.35 and Y2.41 (roughly at the center of the intracellular exposed surface of the receptor) and residue K6.35. (TIF) [file pcbi.1002193.s003.tif]

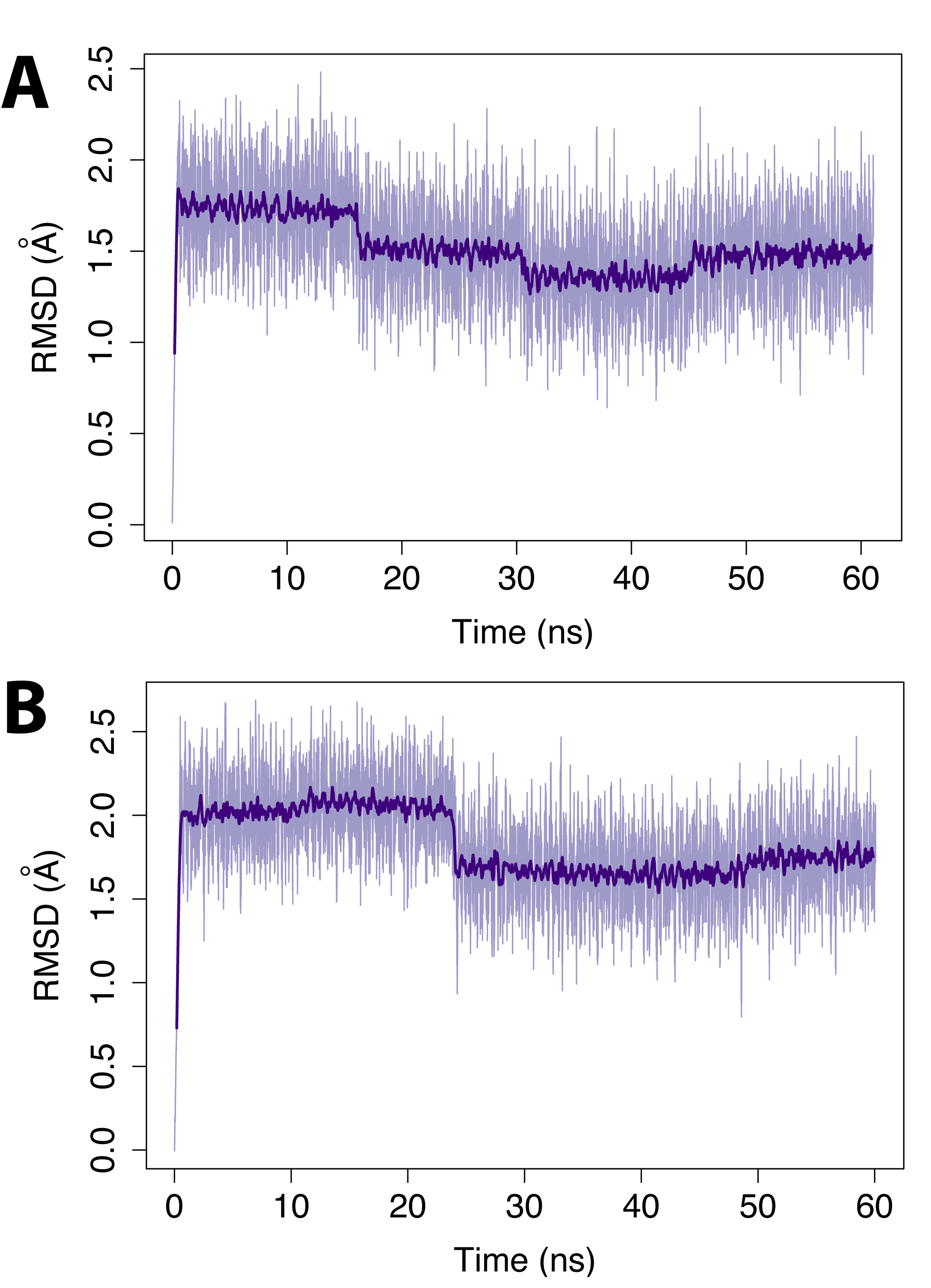

Supplement: Figure S4 — Time evolution of the RMSD of alprenolol and B2AR. RMSD vs. time of (A) the alprenolol heavy atoms after alignment of the B2AR Cα atoms and (B) the B2AR Cα atoms with respect to the initial conformation. The initial structure was extracted from the s∼0.2 and z∼0.0 Å basin in figure S3. (TIF) [file pcbi.1002193.s004.tif]

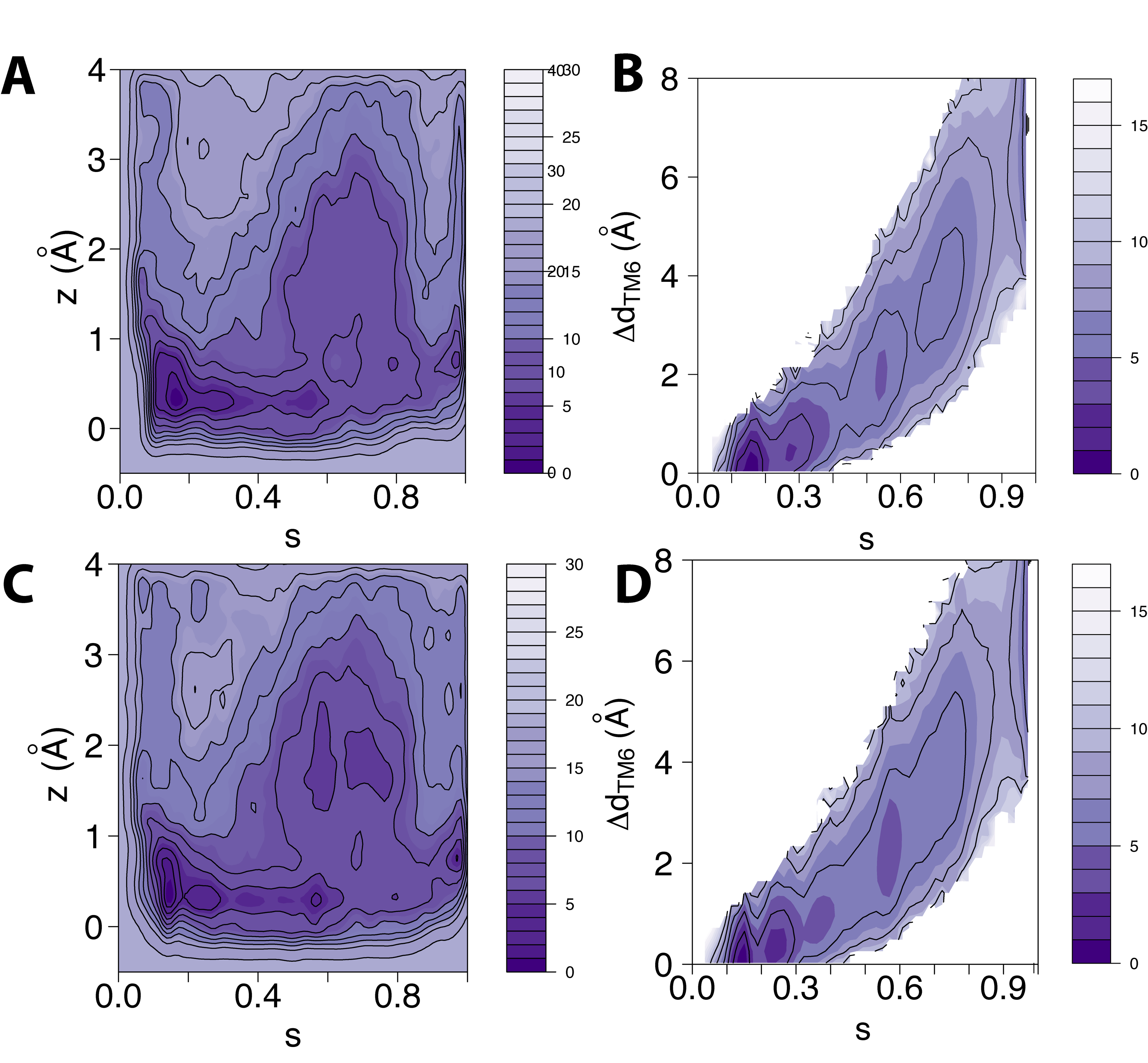

Supplement: Figure S5 — Additional analyses of the simulations of the B2AR bound to the inverse antagonists carazolol and ICI- 118,551 . (A and C) Free-energy of carazolol- and ICI-118,551-bound B2AR as a function of the position along (s) and the distance from (z) the activation pathway. The surface has been shifted so that the lowest energy minima correspond to reference free-energy values; contours are spaced by 2 kcal/mol. (B and D) Free-energy projection as a function of the path variable s and the displacement of TM6 for the inverse agonists carazolol- and ICI-118,551-bound B2AR, respectively. The latter is defined by the distance between the midpoint of an imaginary line connecting residues K6.35 and Y2.41 (roughly at the center of the intracellular exposed surface of the receptor) and residue K6.35. (TIF) [file pcbi.1002193.s005.tif]

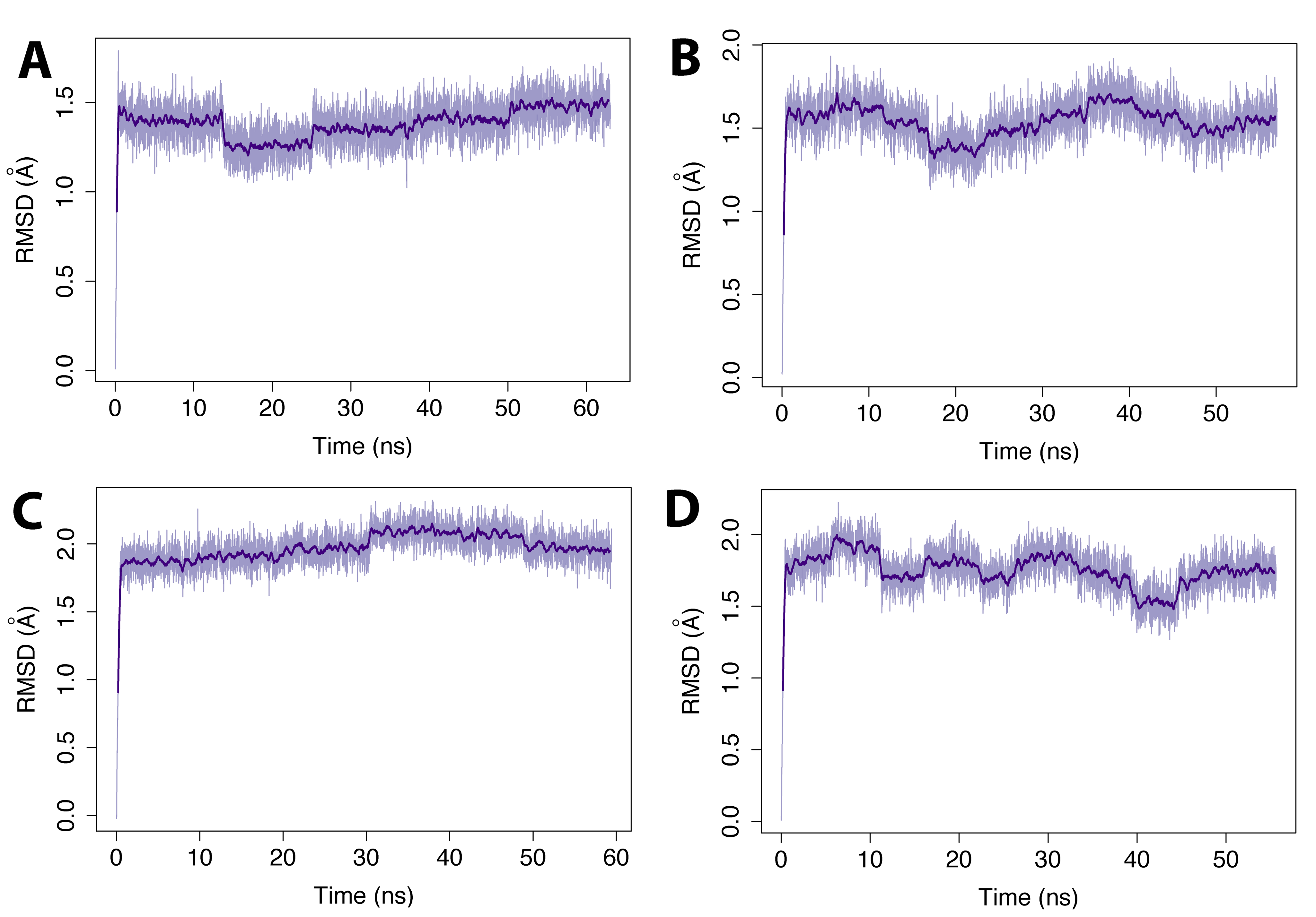

Supplement: Figure S6 — Time evolution of the RMSD of carazolol, ICI- 118,551 , and B2AR. RMSD vs. time of the (A) carazolol and (B) ICI-118,551 heavy atoms after alignment of the B2AR Cα atoms, and the B2AR Cα atoms in the simulations with (C) carazolol or (D) ICI-118,551 with respect to the initial structure. The initial structure was extracted from the s∼0.2 and z∼0.0 Å basins in Figure S5. (TIF) [file pcbi.1002193.s006.tif]

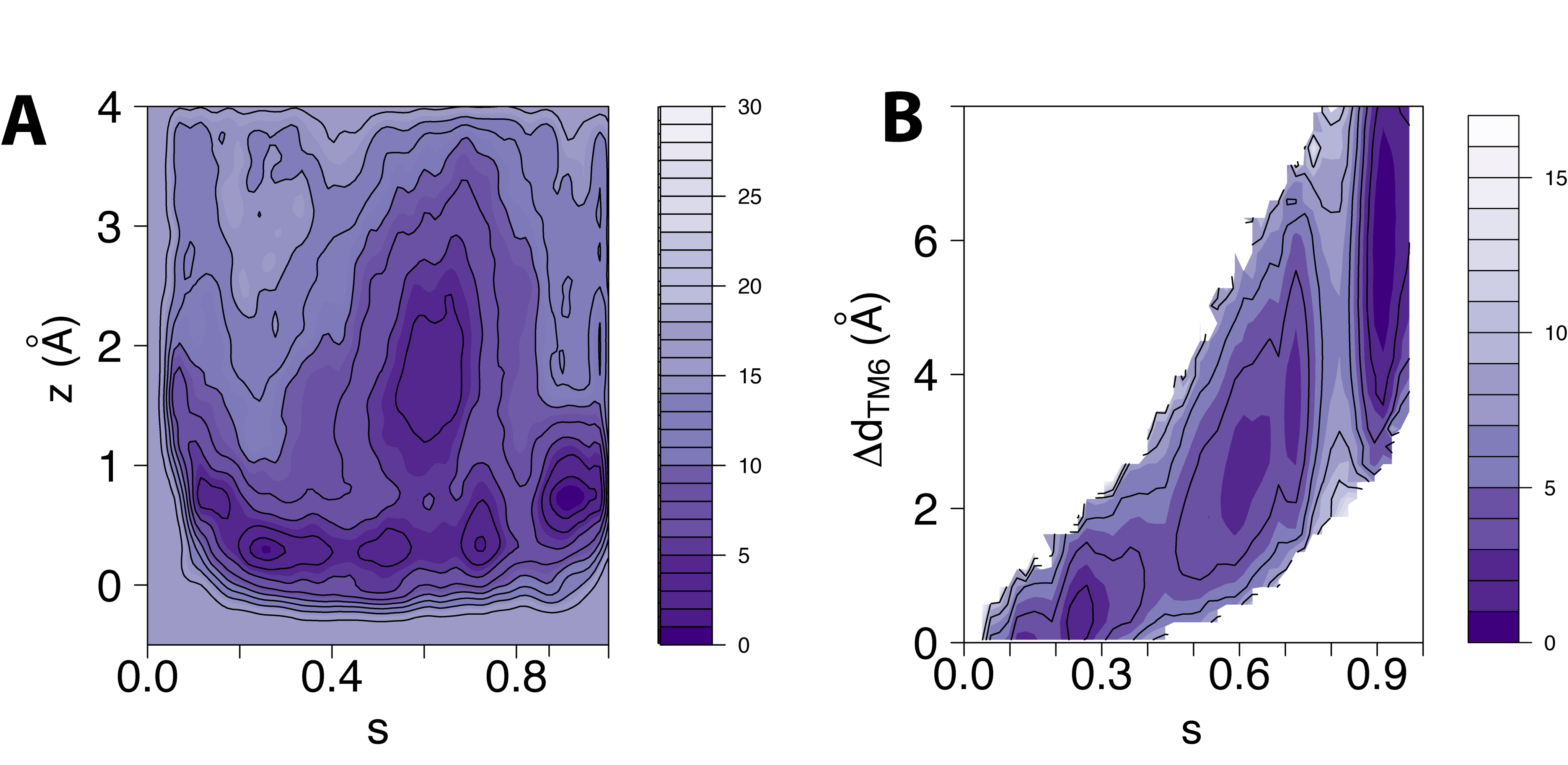

Supplement: Figure S7 — Additional analyses of the simulations of the B2AR bound to the full antagonist epinephrine. (A) Free-energy of the full agonist epinephrine-bound B2AR as a function of the position along (s) and the distance from (z) the activation pathway. The surface has been shifted so that the lowest energy minima correspond to reference free-energy values; contours are spaced by 2 kcal/mol. (B) Free-energy projection as a function of the path variable s and the displacement of TM6. The latter is defined by the distance between the midpoint of an imaginary line connecting residues K6.35 and Y2.41 (roughly at the center of the intracellular exposed surface of the receptor) and residue K6.35. (TIF) [file pcbi.1002193.s007.tif]

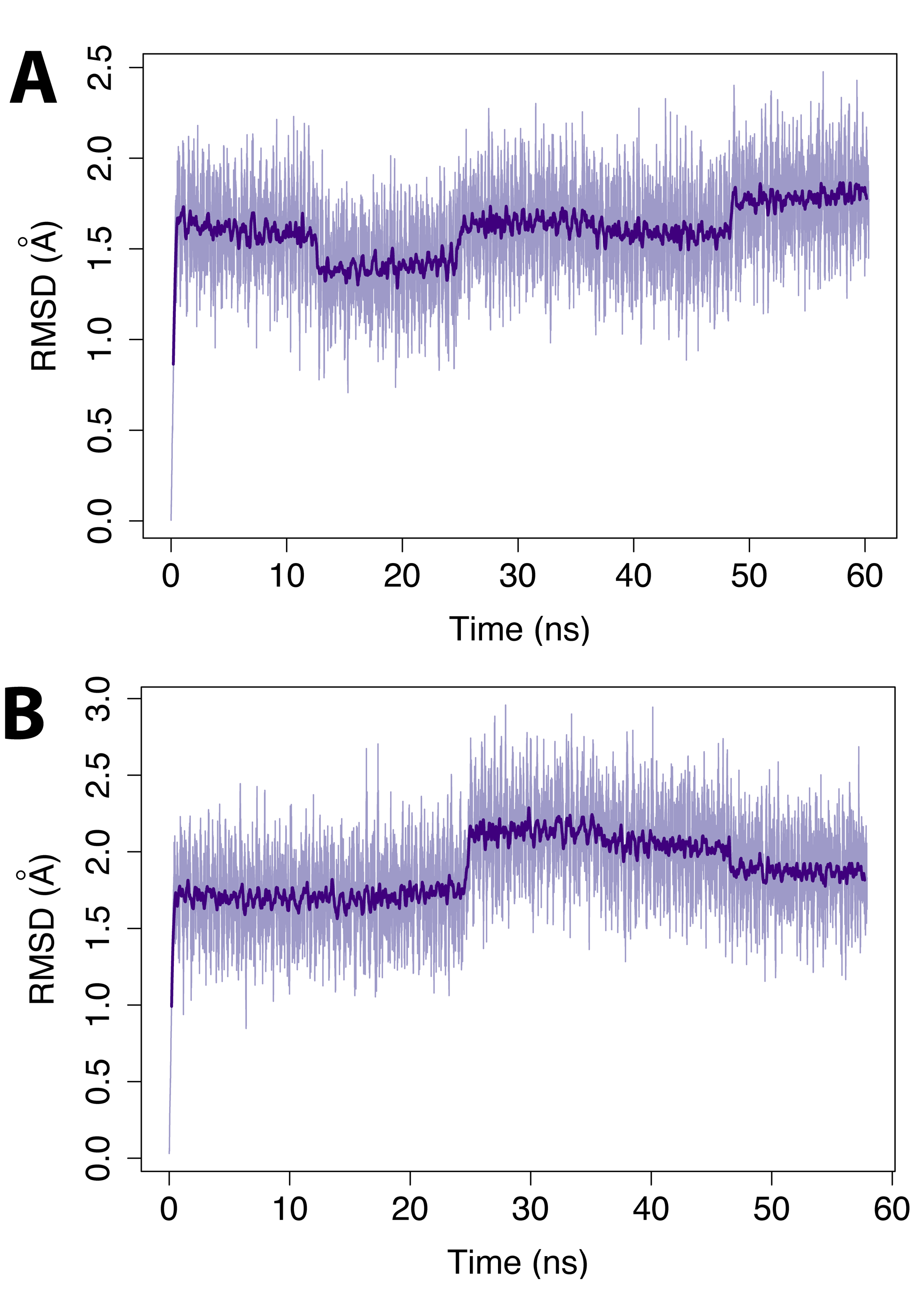

Supplement: Figure S8 — Time evolution of the RMSD of epinephrine and B2AR. RMSD vs. time of (A) the epinephrine heavy atoms after alignment of the B2AR Cα atoms, and (B) the B2AR Cα atoms with respect to the initial structure. The initial structure was extracted from the s∼0.9 and z∼0.5 Å basins in Figure S7. (TIF) [file pcbi.1002193.s008.tif]

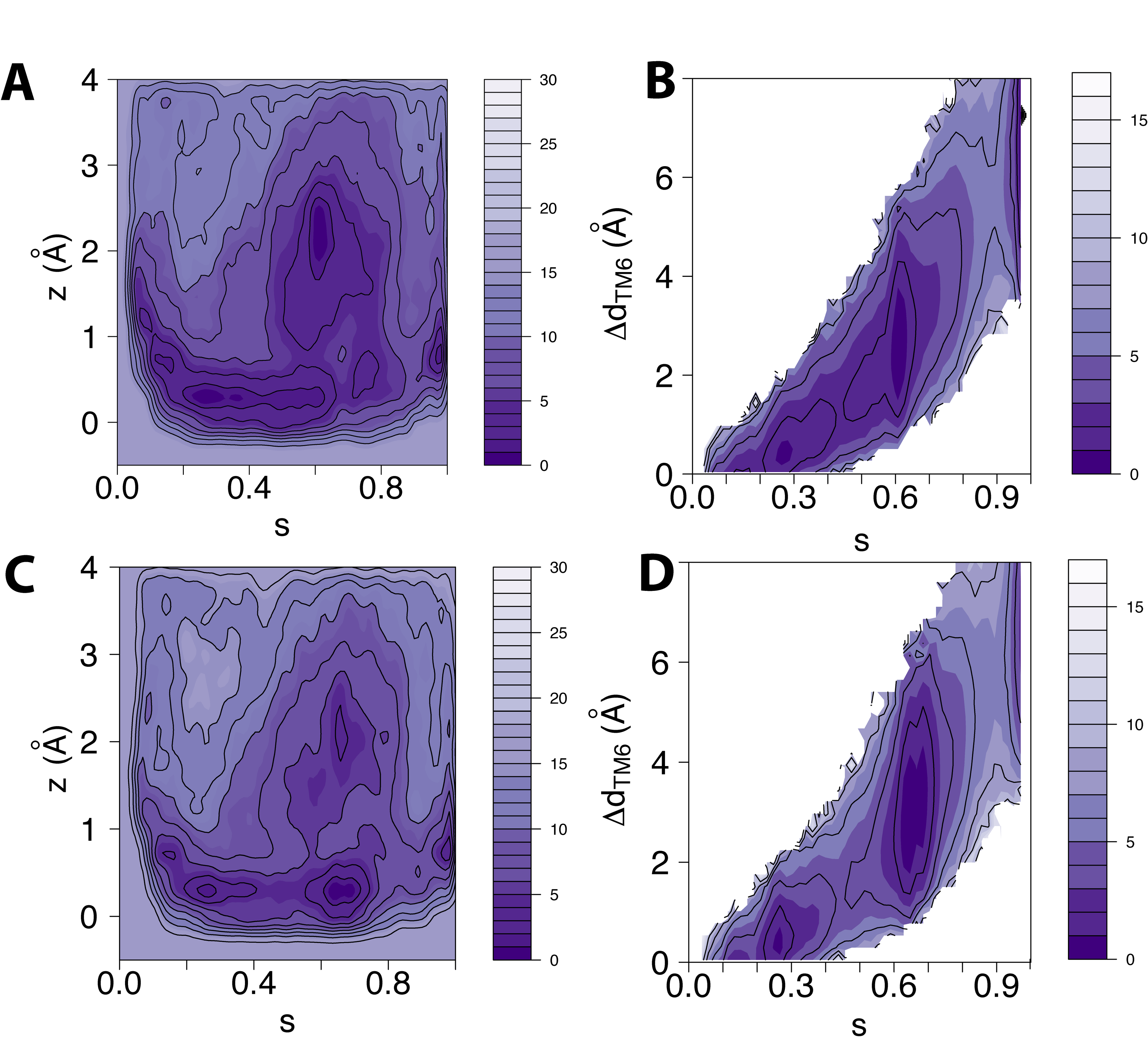

Supplement: Figure S9 — Additional analyses of the simulations of the B2AR bound to the partial agonists catechol and dopamine. (A and C) Free-energy of the partial agonists catechol- and dopamine-bound B2AR as a function of the position along (s) and the distance from (z) the activation pathway. The surface has been shifted so that the lowest energy minima correspond to reference free-energy values; contours are spaced by 2 kcal/mol. (B and D) Free-energy projection as a function of the path variable s and the displacement of TM6 for the partial agonists catechol- and dopamine-bound B2AR, respectively. The latter is defined by the distance between the midpoint of an imaginary line connecting residues K6.35 and Y2.41 (roughly at the center of the intracellular exposed surface of the receptor) and residue K6.35. (TIF) [file pcbi.1002193.s009.tif]

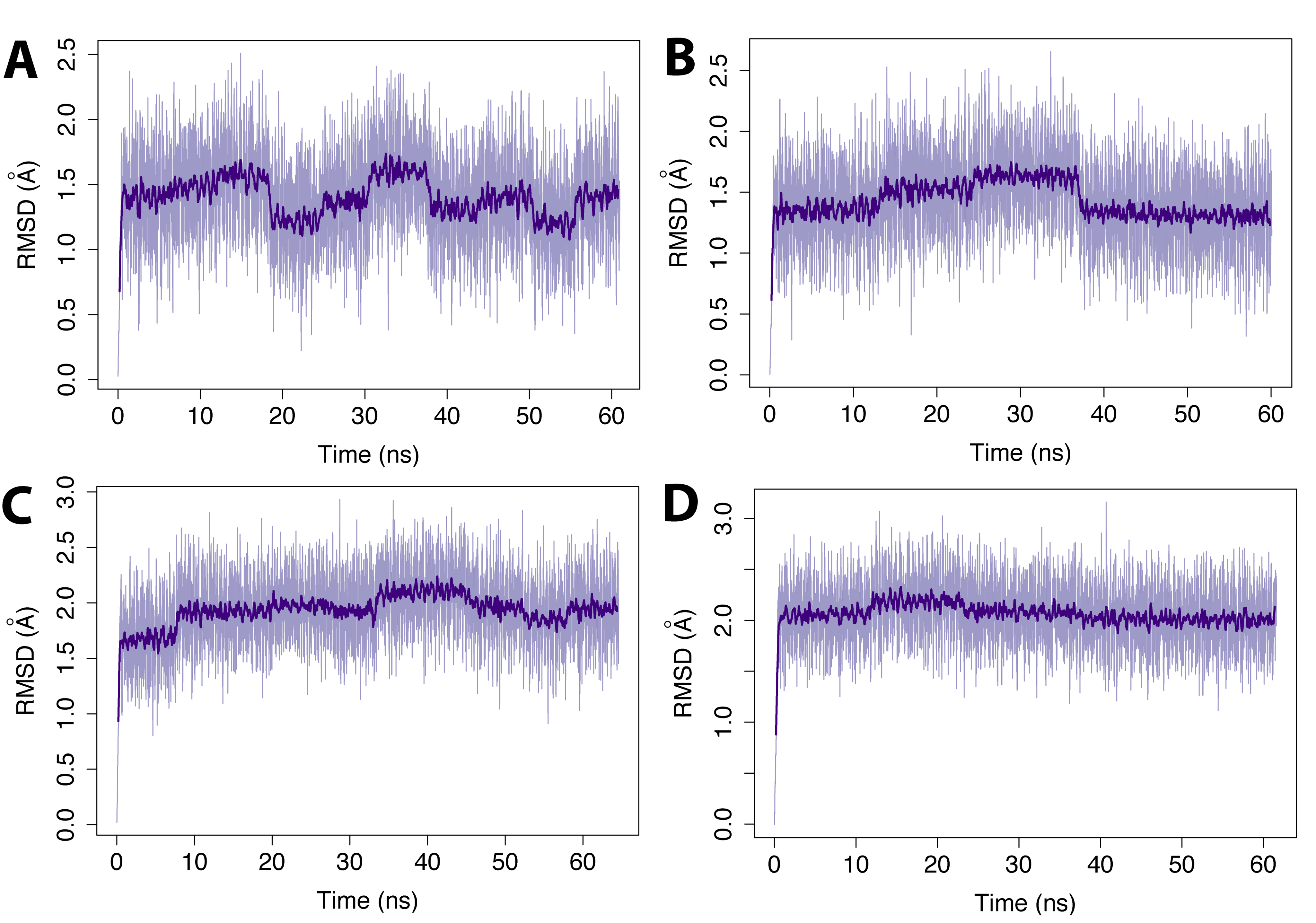

Supplement: Figure S10 — Time evolution of the RMSD of catechol, dopamine, and B2AR. RMSD vs. time of the (A) catechol and (B) dopamine heavy atoms after alignment of the B2AR Cα atoms, and of the B2AR Cα atoms in the simulations with (C) catechol or (D) dopamine with respect to the initial structure. The initial structure was extracted from the s∼0.6 basins in Figure S9. (TIF) [file pcbi.1002193.s010.tif]
