# Supplementary material for: Ligand-Induced Modulation of the Free-Energy Landscape of G Protein-Coupled Receptors Explored by Adaptive Biasing Techniques
Source: PLoS Comput Biol. 2011 Oct 13;7(10):e1002193. doi: 10.1371/journal.pcbi.1002193 (PMC3192824; doi:10.1371/journal.pcbi.1002193)
Supplement: Table S1 — List of the production runs performed in this study. Number and length of independent simulations carried out for each system. (DOC) [file pcbi.1002193.s011.doc]

**Table S1**

|  | # of independent simulations | Length |
| --- | --- | --- |
| **Adiabatic biased MD** |  |  |
| Unliganded B2AR | 10 | ~5 ns each |
|  |  |  |
| **Metadynamics** |  |  |
| Unliganded B2AR | 1 | 300 ns |
| Carazolol-bound B2AR | 1 | 300 ns |
| ICI-*118-551*-bound B2AR | 1 | 300 ns |
| Aplrenolol-bound B2AR | 1 | 300 ns |
| Dopamine-bound B2AR | 1 | 300 ns |
| Catechol-bound B2AR | 1 | 300 ns |
| Epinephrine-bound B2AR | 1 | 300 ns |
|  |  |  |
| **Unbiased MD** |  |  |
| Unliganded B2AR | 1 | 50 ns |
| Carazolol-bound B2AR | 1 | 50 ns |
| ICI-*118-551*-bound B2AR | 1 | 50 ns |
| Aplrenolol-bound B2AR | 1 | 50 ns |
| Dopamine-bound B2AR | 1 | 50 ns |
| Catechol-bound B2AR | 1 | 50 ns |
| Epinephrine-bound B2AR | 1 | 50 ns |
